# Shared Inflammatory Pathology of Stroke and COVID-19

**DOI:** 10.3390/ijms23095150

**Published:** 2022-05-05

**Authors:** Kathryn E. Sánchez, Gary A. Rosenberg

**Affiliations:** 1 Center for Memory and Aging, University of New Mexico, Albuquerque, NM 87106, USA; KaESanchez@salud.unm.edu; 2 Department of Neurology, University of New Mexico, Albuquerque, NM 87106, USA

**Keywords:** COVID-19, stroke, matrix-metalloproteinases, blood-brain barrier, endothelial cells, astrocytes

## Abstract

Though COVID-19 is primarily characterized by symptoms in the periphery, it can also affect the central nervous system (CNS). This has been established by the association between stroke and COVID-19. However, the molecular mechanisms that cause stroke related to a COVID-19 infection have not been fully explored. More specifically, stroke and COVID-19 exhibit an overlap of molecular mechanisms. These similarities provide a way to better understand COVID-19 related stroke. We propose here that peripheral macrophages upregulate inflammatory proteins such as matrix metalloproteinases (MMPs) in response to SARS-CoV-2 infection. These inflammatory molecules and the SARS-CoV-2 virus have multiple negative effects related to endothelial dysfunction that results in the disruption of the blood–brain barrier (BBB). Finally, we discuss how the endothelial blood–brain barrier injury alters central nervous system function by leading to astrocyte dysfunction and inflammasome activation. Our goal is to elucidate such inflammatory pathways, which could provide insight into therapies to combat the negative neurological effects of COVID-19.

## 1. Introduction

**Figure 1:**
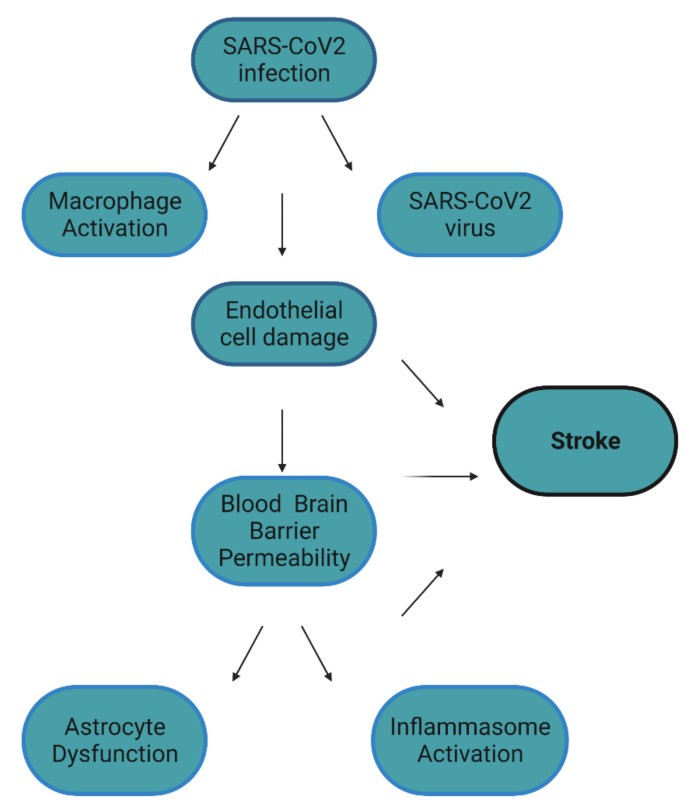
Factors that contribute to stroke in COVID-19. It is hypothesized that COVID-19 directly and indirectly causes endothelial cell damage. This damage results in blood–brain permeability, which results in astrocyte dysfunction and inflammasome activation in the CNS. All these factors, especially endothelial cell damage, can contribute to the development of COVID-19 related stroke. Figure created with BioRender.

Though peripheral symptoms are well characterized in COVID-19, neurological symptoms remain to be further understood. Such symptoms include anosmia, headaches, seizures, depression, and stroke [1,2,3]. Stroke can have profound implications as it is the leading cause of disability worldwide and results in 5.5 million deaths annually [4,5]. In the context of COVID-19, an increased risk of stoke was first established in preliminary studies. More specifically, 5% of COVID-19 patients displayed acute cerebrovascular disease [3]. In additional studies, COVID-19 patients presented a 5% risk of ischemic stroke, 0.5% risk of cerebral venous sinus thrombosis, and a 0.5% risk of cerebral hemorrhage [6]. When this association was further studied in larger populations, 0.9% of COVID-19 patients had a stroke. Of these events, 79% were ischemic stroke, 17% intracerebral/subarachnoid hemorrhage, and 4% cerebral sinus thrombosis [7]. Though these epidemiolocal studies established an association between stroke and COVID-19, only a few studies have speculated on the molecular mechanisms leading to COVID-19 related stroke. We propose here a novel molecular mechanism that involves biomarkers shared by COVID-19 and stroke (Table 1). COVID-19 infection results in the elevation of protein release by macrophages such as matrix-metalloproteinases (MMPs). We hypothesize that both MMPs and the SARS-CoV2 virus both damage endothelial cells. Endothelial cell dysfunction and subsequent BBB permeability lead to further nervous system damage and stroke (Figure 1).

## 2. MMPs

### 2.1. COVID-19 and MMPs

MMPs are a pathological hallmark of many neurodegenerative diseases and are upregulated in both stroke and COVID-19 [19,20,21,22,23]. MMPs have recently been considered in the context of COVID-19 [24]. For example, MMPs have been detected in the periphery of COVID-19 patients. One such study evaluated patients that displayed respiratory failure, and found these patients had elevated MMP-9 in their serum [25]. Notably, this elevation of MMP-9 is long-term, as MMP-9 is detected three months after infection [26]. MMP-9 is also elevated in patients with high risk of hospitalization such as those that are diabetic and obese [8]. The source of this MMP-9 is likely to be neutrophils and macrophages of the lung [27,28]. More recently, the implications of MMP-9 upregulation in COVID-19 have been solidified. For example, it causes cardiac endothelium barrier dysfunction [29]. Another MMP that was elevated in the serum of COVID-19 patients is MMP-3 (stromelysin-1) [30]. In fact, the impact of MMP-3 was confirmed in other studies related to respiratory inflammation. In one investigation, a lipopolysaccharide (LPS) induced model of respiratory distress showed that MMP-3 was elevated. More importantly, its elevation was reversed through pharmacological inhibition [31]. MMP-8 is also relevant to COVID-19, as its target (triggering receptors expressed on myeloid cells-1) TREM1 correlates with the severity of COVID-19 patients [32]. The molecular link between MMPs and SARS-CoV-2 has been hypothesized as being mainly based on respiratory and pulmonary stress [33]. In the lung, metalloproteinases are derived from inflammatory cells (such as neutrophils, alveolar macrophages, and eosinophils) and parenchymal cells (such as pulmonary endothelial cells, type II epithelial cells, and interstitial fibroblasts) [33]. In other studies related to lung damage, similar to COVID-19, MMP-1, MMP-2, MMP-8, MMP-9, MMP-12, and MMP-14 contribute to COPD-associated pulmonary damage; MMP-1, MMP-2, MMP-3, MMP-7, MMP-8, MMP-9, MMP-12, MMP-14, and MMP-25 are associated with asthma; MMP-1, MMP-2, MMP-3, MMP-7, MMP-8, MMP-9, MMP-11, MMP-12, and MMP-13 contribute to acute lung injury (ALI) and acute respiratory distress syndrome (ARDS); and MMP-1, MMP-2, MMP-3, MMP-7, MMP-8, and MMP-9 contribute to idiopathic pulmonary fibrosis [34,35,36,37,38,39].

### 2.2. Stroke and MMPs

One example of how MMPs have been involved in BBB permeability is stroke. More specifically, clinical studies revealed higher levels of MMPs in stroke patients. For example, MMP-9 was found to be elevated in the serum of patients that displayed myocardial infarction [9]. MMP-9 was also elevated in patients with acute ischemic stroke compared to controls. MMP-9 levels also correlated with larger infarct volume, increasing severity of stroke, and poor functional outcome [40]. A wide range of cell types have been shown to express MMP-9 following stroke including neurons, microglia, and endothelial cells [41]. In vivo, the inhibition of MMPs also prevents the cleavage of tight junction proteins relevant to stroke damage in a rodent model [42,43].

Taken together, these studies establish that MMPs are present in the periphery of both stroke and COVID-19 patients. The relevance of an increase in MMP activity during COVID-19 has not been thoroughly explored, but its impact can be theorized due to other models of inflammation. There is preliminary evidence to suggest this, since in vitro MMP-9 is upregulated by endothelial cells in response to SARS-CoV-2 [44]. As a result, these MMPs can damage endothelial cells.

## 3. Endothelial Cells

### 3.1. Endothelial Cell Damage in Stroke

Numerous studies have investigated how endothelial cell injury contributes to stroke. In vitro, endothelial cells have been used to study the impact of proteins that arise from vascular damage. For example, immortalized endothelial bEnd3 cells were challenged with hemin and subsequently evaluated for apoptosis. It was found that hemin exposure increased the release of cleaved caspase-3, supporting the idea that endothelial apoptosis may decrease blood flow in the damaged capillary and enhance its leakage [45]. Endothelial cell death is often associated with a disruption of the BBB [45,46]. Notably endothelial cell death may also contribute to BBB breakdown. This theory has been investigated with electron microscopy. Endothelial layer integrity was compromised in brain areas with FITC-albumin extravasation into the neuropil [47,48]. In vivo, there is also evidence that stroke damages the tight junction proteins of the BBB. In a rodent model of stroke, tight junction proteins such as zona occludens-1 (ZO-1), Claudin-5, and Occludin are compromised [42,43,49].

### 3.2. Endothelial Cell Damage in COVID-19

Similar to stroke, endothelial cells may be impacted by COVID-19 [50,51]. It is theorized that endothelial cells may be vulnerable to SARS-CoV-2 due to past research on viral infection. For example, HIV causes loss of BBB integrity [52]. More importantly, it has recently been reported that SARS-CoV-2 can infect choroid plexus cells in numerous models [28,29]. Such in vitro studies have shown compelling evidence of transcellular transport. Human endothelial cells were infected with COVID-19 in transwell assays [1]. When SARS-CoV-2 was used to infect the cells from the apical side, viral RNA was detected in cells on the basal side [1]. Taken together, these studies indicate that endothelial cells can become infected with the virus. There are many possible mechanisms of the viral entry into endothelial cells, but candidate receptors include the angiotensin converting enzyme 2 (ACE2) receptor and neuropilin-1 (NRP1). SARS-CoV2 is well-known to bind to the ACE2 receptor to gain entry into cells, which can negatively impact blood pressure, electrolyte homeostasis, vascular and cardiac remodeling, and inflammation [53,54]. Notably, ACE2 is also abundant in endothelial cells, making it a likely candidate for viral entry into endothelial cells. Though CD147 (Basigin) has been viewed as an alternative source of entry in endothelial cells, recent findings have shown that recombinant forms of the SARS-CoV-2 spike protein do not interact with CD147 when investigating human cells [54,55]. One last candidate receptor includes NRP1, which is also expressed heavily by endothelial cells. Though not as well established as ACE2, NRP1 directly binds the furin-cleaved S1 fragment of the spike protein [56]. Further corroborating its importance, this interaction is reduced by a small-molecule inhibitor or monoclonal antibodies that reduces viral infection in cell culture. The presence of the SARS-CoV-2 spike protein in the endothelial cells of small CNS vessels was confirmed in histological studies of COVID-19 patients [57,58]. Though the precise mechanism of COVID-19 entry into the CNS has not been confirmed, theoretical studies imply that COVID-19 interacts with tight junction proteins. On the other hand, there are data suggesting that the basal lamina is compromised by COVID-19 [57]. However, not all tight junction proteins during COVID-19 have been investigated. For example, no data to date have been collected on how COVID-19 impacts cingulin. Cingulin is of particular importance since it colocalizes with ACE2, the previously mentioned receptor that plays a role in COVID-19 entry into a cell [59]. Cingulin was also found to be downregulated in COVID-19 patients [60].

Matrix metalloproteinases (MMPs) have been investigated in endothelial damage caused by the SARS-CoV-2 virus. Gelatinases MMP-2 and -9 contribute to inflammation of the lungs [61]. In fact, endothelial cells, which release MMPs under inflammatory conditions, are implicated in airway damage [62]. Such MMPs include MMP-1, MMP-2, MMP-9, and MT1-MMP [63,64,65]. There is evidence that MMPs are critical to the inflammatory cytokine storm observed in COVID-19. Their involvement has been established in preliminary investigations with patient samples. For example, MMP-9 and its MMP-9/NGAL heterodimer were significantly increased in the plasma of COVID-19 patients compared to healthy controls [27].

Taken together, these studies establish that there is likely endothelial damage that occurs due to the presence of MMPs in COVID-19 patients. They also emphasize that the virus is capable of directly damaging endothelial cells. This damage is evident in patient samples, where vascular endothelial growth factor (VEGF) is elevated just as it is in stroke [10,11]. These investigations solidify the role of endothelial injury in both stroke and COVID-19. Endothelial cell damage is apparent in both, as VEGF is elevated in serum samples of patients with acute ischemic stroke and COVID-19. Therefore, it is likely that the BBB is compromised during a COVID-19 infection.

## 4. Blood–Brain Barrier

Although the BBB is thought to make the CNS an immune privileged site, there is an interchange of molecules and cells. The BBB restricts entry of large, charged molecules, but allows limited entry of small molecules such as water, and offers no restriction to gases. Under normal conditions, this interchange is minimal, but it can be greatly increased by trauma, ischemic injury, and infection. Most drugs are prevented from entering the brain except for those that are lipid soluble including anesthetic gases [23,42,66,67,68]. The BBB is formed by a series of cell layers beginning with the endothelial cells that form the first line of defense offered by the tight junction proteins that “zip lock” the cells together, forming epithelial-like sheets with high electrical resistance [49,66,69]. In the face of inflammatory insults, these tight junction proteins become vulnerable. In an LPS induced model of neuroinflammation, the highest dose of LPS (3 mg/kg) administered to mice produced a significant increase in BBB permeability [70]. These initial findings were corroborated with a TNFα injection model [71]. In some investigations, mice that displayed an upregulation of this proinflammatory cytokine also exhibited altered tight junction protein integrity [72]. Taken together, these studies emphasize how general inflammation can degrade tight junction proteins of the BBB.

## 5. The Inflammasome

### 5.1. The Inflammasome and Endothelial Cells

Inflammasome activation is a crucial aspect of inflammation. More specifically, inflammasome activation in the case of a viral infection may drive the susceptibility of patients with preexisting metabolic complications worsened by age [73]. In the CNS, inflammasome activity is apparent in numerous neurological diseases such as COVID-19 and stroke [74,75,76,77,78]. In neurodegenerative diseases, pathogenic proteins can stimulate an immune response. These pathogen associated molecular patterns (PAMPS) and danger associated molecular patterns (DAMPS) cause the innate immune system of the central nervous system to react in a damaging manner [79,80,81,82,83,84]. For example, microglia express pattern recognition receptors (PRRs) such as Toll-like receptors (TLRs) and Nod-like receptors that are responsible for responding to DAMPS and PAMPS. As a result of PRR activation, inflammatory signaling cascades are triggered [85,86,87,88,89,90]. One such example of an inflammatory signaling cascade is the inflammasome pathway [91]. The (NACHT, LRR, and PYD domain-containing protein 3) (NLRP3) inflammasome is defined as a multiprotein complex with the following core proteins: three domains of NLRP3; the adaptor protein apoptosis-associated speck-like protein containing a CARD (ASC); and inflammatory caspase 1 (cysteine-dependent aspartate-directed protease 1) [89,92,93,94]. The ASC and pro-caspase 1 components of the complex promotes the activation of caspase 1 and the processing of cytoplasmic targets including IL-1β and IL-18 [77,95,96,97,98]. Activation of the inflammasome is regulated by a two-step process that begins with priming. Priming allows for transcriptional upregulation of the NLRP3 genes in response to the recognition of pathogen-associated molecular patterns (PAMPs) such as lipopolysaccharides and viral RNA, or damage-associated molecular patterns (DAMPs) such as ATP and reactive oxygen species (ROS) [77,96,97]. Furthermore, the inflammasome complex can regulate other processes in the cell such as gene regulation and transcription. Because of inflammasome activation, the prolonged elevation of cytokines can have negative consequences for neurons. Previous research has established the upregulation of inflammasomes in diseases such as Parkinson’s disease, Alzheimer’s disease, and stroke [76,90,99]. In fact, in a model of stroke induced by carotid artery thrombosis, components of the inflammasome were increased. This increase in proteins associated with the inflammasome such as caspase-1, NLRP1, and ASC was reversed by inhibiting the activity of NLRP1 [100].

It is notable that proteins in the inflammasome complex are expressed by numerous cell types of the CNS. This includes the innate immune cells of the CNS and microglia as well as other cells such as endothelial cells and astrocytes [101,102,103,104]. By better understanding the role of the inflammasome at a cell specific level, we will be better equipped to target inhibitors [105].

Inflammasomes in microglia, which are innate immune cells of the CNS, have been extensively reviewed. Though there is not currently a full understanding of the microglial inflammasome in the CNS during COVID-19 infection, the inflammasome in ischemic stroke is well understood. The activation of the inflammasome in microglia during stroke is likely due to a change in ion flux [106,107,108]. ROS, potassium efflux, cathepsins, and DAMPs could all be sensed by appropriate inflammasome sensors, leading to activation of the inflammasome [109,110,111,112].

Though typically associated with immune cells, inflammasome machinery and adaptor proteins are also expressed by the endothelium. For example, primary human bronchial epithelial cells were evaluated after exposure to toxic doses of crystalline silica for changes in NLRP3, caspase-1, and IL-1β expression. In response to the toxicant, the cells exhibited a transcriptional and translational upregulation of the components of the NLRP3 inflammasome, and more importantly, the activation of caspase-1 [113]. The role of the inflammasome was solidified in rodent models. For example, following chronic hypoxia, mice lacking the inflammasome adaptor, ASC, were protected from elevated right ventricular systolic pressure (RVSP) and right ventricular hypertrophy [114]. The *ASC*^−/−^ mice failed to show a pulmonary increase in caspase-1, IL-18, or IL-1β, in contrast to wild type controls [114]. While this research was conducted in a rodent model, it is notable that this work established that oxygen deprivation, a critical part of COVID-19 injury, may cause inflammasome activation. Inflammasome activation is apparent in COVID-19 patients, and in one case study, IL-1β was even detected in the CSF of a patient [13]. The findings of this study were corroborated in another investigation in which other inflammasome proteins were elevated in COVID-19 patient samples [16,17,18].

### 5.2. The Inflammasome and Astrocytes

In an inflammatory state such as during COVID-19 infection and stroke, astrocytes can no longer perform functions that are important to neuronal survival and homeostasis [115]. For example, in an ischemic state, astrocytes increase glial fibrillary acidic protein and undergo rapid proliferation and swelling [14,15,116]. They also alter ACE2 expression in both COVID-19 and stroke, solidifying the role of the receptor in vascular stress [66,117,118]. While the inflammasome has been primarily studied in the context of microglia, it is crucial to consider that other cells of the CNS that are important to BBB function also express its components. For example, astrocytes express NLRP3 as well as NLRP2, ACS, and caspase-1 during CNS insults [119,120,121]. More specifically, these studies showed that astrocytes are crucial in the activation of the inflammasome during CNS inflammation. In the MPTP mouse model of Parkinson’s disease, caspase-1 expression increased IL-1β in astrocytes. It was established that the use of a dopaminergic receptor agonist suppressed components of the inflammasome in astrocytes [119]. Astrocytes were also investigated in the context of systemic inflammation with lysophosphatidylcholine (LPC) serving as the inflammatory agent. Exposure of primary astrocytes, lacking the NLRP3 gene, to LPS, failed to release IL1-β, establishing astrocytes as a source of inflammasome activation [120]. In the context of stroke, astrocytes also contribute to inflammasome activation. In a study using primary cell culture and oxygen deprivation, astrocytes exposed to hypoxia exhibited signs of apoptosis and cell injury. More specifically, the protein levels of caspase-12, cleaved caspase-3, NLRP3 inflammasome components, and IL-1β were significantly elevated. These changes were reversed using the caspase inhibitor Z-ATAD-FMK [121]. Taken together, these studies emphasize the role that astrocytes may play in the inflammatory response to stroke.

## 6. Anti-Inflammatory Agents to Target COVID-19

As the COVID-19 pandemic develops, numerous targets of inflammation should be considered. These include inhibitors of the inflammasome, MMPs themselves, and MMP cleavage targets.

### 6.1. Inhibitors of the Inflammasome

In a model of multiple sclerosis, the inflammasome was inhibited by VX-765 (Belnacasan), a selective inhibitor of caspase-1. Though this has only been tested in small clinical trials, VX-765 has also been shown to be effective in a mouse model of Alzheimer’s disease [105,122,123,124]. In J20 mice, knocking out caspase-1 improved microglia mediated inflammation, decreased amyloid-β accumulation, and prevented memory deficits [125,126]. In the case of synucleinopathies, both in vitro and in vivo work support the use of VX-765 [127,128]. In vitro, VX-765 prevented the activation of the inflammasome in neuronal BE(2)-M17 cells overexpressing α-synuclein after stimulation with known inflammasome activators [128]. In vivo, a mouse model of multiple systems atrophy was used to evaluate VX-765. In this transgenic mouse, α-synuclein was overexpressed, but VX-765 reduced the load of α-synuclein. It also prevented the loss of dopaminergic neurons in the substantia nigra. Due to its success in diseases of the CNS, VX-765 has also been used in stroke animal models. More specifically, mice with a middle cerebral artery occlusion were treated with VX-765. These mice displayed a smaller infarct volume and less neurological impairment compared to the untreated controls. When they evaluated the molecular mechanisms that led to the improvement, they determined that NFκB nuclear translocation was inhibited in microglia by VX-765 [129]. The option of using VX-765 as a therapeutic is ideal given that it can penetrate the BBB and is nontoxic. Furthermore, it has been used in a clinical trial in which it was determined to be safe [130,131]. More recently, in an experimental trial, the anti-inflammatory drug Colchicine was investigated in the context of COVID-19. Through inhibition of the inflammasome, it was found that Colchicine treated patients had reduced markers of vascular damage [78]. Notably, these patients also displayed a slower clinical deterioration, making this a promising pharmaceutical target in the future.

### 6.2. Inhibitors of MMP Cleavage Targets

MMPs are also a promising therapeutic target in the context of long-haul neurological complication of COVID-19 due to their alteration in stroke and depression. Anxiety and depression are particularly relevant since they are the more common comorbidities of infection. In fact, 42% of long-haul patients reported both symptoms after having COVID-19 [132]. In addition, MMPs have been linked to neuropsychiatric disorders [133,134,135]; they are elevated in major depression compared to healthy controls [136]. Understanding the link between MMPs and neurological complications to COVID-19 are crucial given how prevalent MMPs are in COVID-19, stroke, and neuropsychiatric disorders. These include MMP-2, MMP-7, and MMP-10 [136]. Targeting MMPs may be able to prevent neurological complications of COVID-19. MMPs have been directly inhibited in the context of COVID-19. For example, a pan-MMP inhibitor doxycycline has been preliminarily investigated in severe cases of COVID-19 [137]. Though MMP inhibition is promising, their cleavage targets may prove more successful since directly inhibiting MMPs has off-target effects [138]. More novel targets of MMPs should be considered. For example, Tenascin C has been upregulated in the bronchoalveolar lavage fluid of severe COVID-19 patients [139]. Tenascin C is a matricellular protein upregulated in numerous cell types during stroke to facilitate central nervous system repair. It can be a component of the glial scar formed in this repair and recovery process [140,141]. Some extracellular matrix proteins are crucial to neuronal survival. For example, there are extracellular matrix proteins such as Brevican and Neurocan that surround the soma of inhibitory neurons in the form of a perineuronal net [142,143]. Brevican and Neurocan, and perineuronal nets, have not yet been evaluated in the context of COVID-19, making them topics of future research. Another MMP target that may be important to consider is the g-protein couple receptor (GPCR) protease-activated receptor 1 (PAR1). PAR1 is a GPCR that has been studied in CNS inflammation. PAR is a seven transmembrane G protein-coupled receptor that is irreversibly proteolytically activated by thrombin. Thrombin binds to and cleaves the N-terminus of PAR1, generating a new N-terminal domain that binds intramolecularly to trigger transmembrane signaling. Activated PAR1 couples to multiple heterotrimeric G protein subtypes including G_12/13_, G_q_, and G_i_ and activates a variety of signaling effectors important for many cellular processes [144,145,146,147]. It is elevated in stroke patients [146,148]. In a mouse model of stroke, it was found that PAR1 agonism decreased infarct volume [146]. Previous research into the link between PAR1 and the inflammasome included the use of an ischemia-reperfusion model of inflammation. Such investigations have found that PAR agonism after injury prevents inflammasome activation [147]. More specifically, it can reduce the production of proteins such as NLRP3, caspase-1, and IL-1β [147,149]. At the cellular level, microglia also respond to PAR1 agonism as they display an inflammatory phenotype that includes the release of IL-1β. Furthermore, PAR1 deficiency prevents the release of IL-1β, implicating its role in mediating inflammasome activation [149,150,151]. Because PAR1 activation can lead to a multitude of inflammatory signaling pathways, it has been under investigated. More specifically, it is underappreciated in its contribution to MMP and inflammasome activation and may therefore be a future pharmaceutical target for COVID-19.

## 7. Conclusions

**Figure 2:**
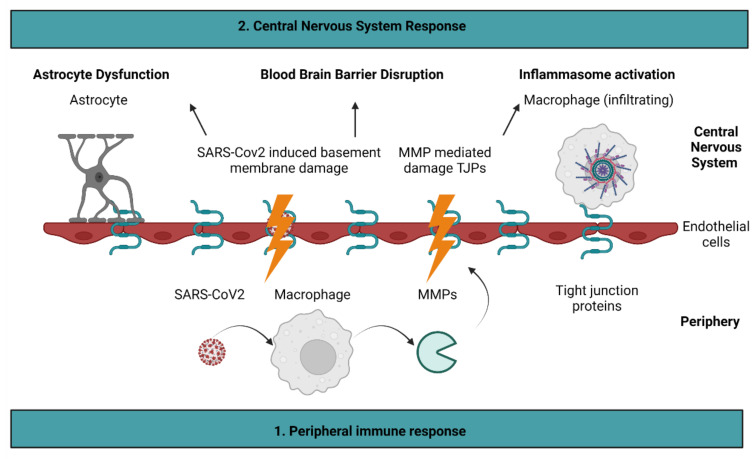
Proposed molecular mechanisms of COVID-19 induced stroke. It is proposed that COVID-19 stimulates the release of matrix metalloproteinases (MMPs) by macrophages. As a result, MMPs cleave tight junction proteins present on endothelial cells. Damage to endothelial cells is exacerbated directly by the SARS-CoV2 virus, which alters basement membrane integrity. Endothelial cell injury leads to blood–brain barrier permeability, which results in astrocyte dysfunction and inflammasome activation. Figure created with BioRender.

Taken together, the recent discovery of stroke as a consequence of COVID-19 infection has been preliminarily explored; MMPs, tight junction proteins, and inflammasome activation all contribute to both stroke and COVID-19. To that end, this review proposes possible molecular mechanisms that can expand the preliminary data already collected on the inflammatory pathways involved in stroke and COVID-19 (Figure 2). To that end, it is proposed that COVID-19 results in the damage of endothelial cells through the upregulation of MMPs and basement membrane injury. This leads to BBB permeability, which results in further inflammation and astrocyte dysfunction. Consequently, stroke may occur. If the molecular mechanisms shared by stroke and COVID-19 are investigated more thoroughly, it is possible that such discoveries will aid in understanding the long-term effects of COVID-19. This understanding will be critical as we begin to emerge from the COVID-19 pandemic.

## Figures and Tables

**Table 1:** The pathological evidence of inflammatory proteins involved in both COVID-19 and stroke.

Biomarker	Control Level [Reported Confidence Interval or S.E.M., or I.Q.R.] (n)	Stroke Level [Reported Confidence Interval, S.E.M., or I.Q.R.] (n)	COVID-19 Level (n) [Reported Confidence Interval, S.E.M., or I.Q.R.]	References ^1^
MMP-9 serum	478.93 ug/L [±134.79] (14) COVID-19 control 78 ng/mL [±59] (35) Stroke control	150 ng/mL [±66] (40)	801.67 ug/L [±155.33] (8)	[8,9]
VEGF serum (endothelial damage)	25.9 pg/mL [12.3, 40.6] (14) COVID-19 control 245 [±80] (26) Stroke control	518 pg/mL [±80] (29)	62.9 pg/mL (45.8, 79.6) (10)	[10,11]
IL-β CSF (inflammasome activation)	0.95 pg/mL [±0.02] (25) Stroke control	30.4 pg/mL [±7.3] (51)	14.8 pg/mL N/A (case study) ^2^ (1)	[12,13]
GFAP plasma (astrogliosis)	141 pg/mL (108–207) (33) COVID-19 control (51) 0 ng/mL [0–0.21] (79)	2.17 ng/mL [0.55–10.12] (34)	206 pg/mL (106–308) (18)	[14,15]

^1^ Unless noted, all studies reported statistically significant differences in disease group biomarkers when compared to proper control groups. Levels reported are either mean or median and are reported here as they were in the original study. Data from the corresponding reference where values obtained is cited in the reference column ^2^ This study was a case study and statistics were not performed. However, other studies have established inflammasome activation in COVID-19 patients [16,17,18].

## Data Availability

Not applicable.
